# The dual role of microglia in Alzheimer’s disease: from immune regulation to pathological progression

**DOI:** 10.3389/fnagi.2025.1554398

**Published:** 2025-03-27

**Authors:** Cong He, Baojiang Chen, Hecai Yang, Xiaoqing Zhou

**Affiliations:** ^1^Second Clinical Medical College, Heilongjiang University of Chinese Medicine, Harbin, China; ^2^First Clinical Medical College, Heilongjiang University of Chinese Medicine, Harbin, China; ^3^Shenzhen Hospital of Beijing University of Chinese Medicine (Longgang), Shenzhen, China

**Keywords:** Alzheimer’s disease, microglia, neuroinflammation, amyloid-beta clearance, genetic variants

## Abstract

Alzheimer’s disease (AD) is a widespread neurodegenerative disorder and one of the major challenges for public health. Despite extensive research, the role of microglia in AD remains complex and dual. The aim of this review is to summarize the most recent advances in research regarding the dual role of microglia in AD concerning both immunomodulation and pathological progression by considering mechanisms of activation of microglia, effects on Aβ clearance, tau pathology, and impacts due to genetic variations on microglial functions. Among these findings are the dual role of microglia, the status of activation for M1 and M2 phenotypes, and the crucial role that genetic variants like TREM2 have in modulating the response of microglia. This review describes how modulation of the microglial signaling pathway might be exploited therapeutically for AD treatment and underlines the relevance of a personalized medicine approach.

## 1 Introduction

Alzheimer’s disease (AD) is a neurodegenerative disease and the most common one; an estimated 6.9 million Americans aged 65 and older live with AD (GBD 2021 US Burden of Disease Collaborators, 2024; Salazar-Londono et al., 2024). If no medical breakthroughs occur in prevention or treatment, the number of people living with AD could double to 13.8 million by 2060. It is estimated that the overall cost of health care, long-term care, and hospice care for people with dementia aged 65 years and older is $360 billion. Health insurance per capita costs for people aged 65 years and older with AD or other dementias is almost three times higher than for people without the disease, and the Medicaid cost is over 22 times higher (Alzheimer’s Association, 2024; Li W. et al., 2024). The challenge of AD is a major public health issue that must be urgently addressed by the public health community around the world. This has turned AD into a global public health challenge.

In recent years, there has been increasing emphasis on the crucial role of microglia in the central nervous system (CNS) during AD pathogenesis with the development of neuroimmunology. As the major immune cells in the CNS, microglia are responsible for maintaining neural environment homeostasis, scavenging pathological proteins, and regulating neuronal function (Is et al., 2024; Jacquet et al., 2024). Recent studies have shown that microglia exert a double role in AD-on one hand, they exert a protective function through immunomodulation and Aβ plaque scavenging; on the other hand, over-activated microglia may trigger chronic inflammatory responses that may further aggravate neuronal damage and pathological development (Onuska, 2020; Long et al., 2022). The mechanism of this dual role has not been fully clarified but is closely related to the regulated expression of multiple AD risk genes carried by microglia, such as TREM2, CD33, etc. (Malik et al., 2015; Liu et al., 2020). Recent advances in genomics and proteomics have further revealed the complex signaling changes of microglia in the development of AD pathology. Large-scale proteomic analyses unraveled early energy metabolic changes associated with the activation of microglia in the brain tissue and cerebrospinal fluid of AD patients and summarized the findings as glial biological patterns underlining the centrality of microglia in AD pathology (Hemonnot et al., 2019; Leng and Edison, 2021; Chen et al., 2023; Zhou X. et al., 2024).

The present review aims to summarize the recent research progress regarding the dual roles of microglia in AD focusing on the correlation between immune modulation and pathological progression. From such a focus, integration of the involved mechanisms into the involved signaling pathways may be able to elucidate the pleiotropic roles microglia may play in AD pathogenesis and therefore provide a new theoretical principle or even potentially research toward a final therapeutic strategy.

## 2 The dual role of microglia in AD

### 2.1 Normal functions of microglia in the brain

As the main immune sensors of the CNS, microglia have highly dynamic morphological and functional properties. In the resting state, microglia assume an astrocytic morphology with abundant dendrites, whose dynamically extending and contracting dendrites are believed to be constantly monitoring the surrounding environment (Pallares-Moratalla and Bergers, 2024). Microglia express a variety of receptors on their surfaces, capable of sensing signaling molecules generated by neuronal activity. Abnormal signals or pathological stimuli rapidly transform microglia from the resting to the activated state. Morphology changes as they migrate to the site of injury and release a variety of cytokines and chemokines (Woodburn et al., 2021). Microglia control neuronal functions through the regulation of cerebral vascular tone, hence local cerebral blood flow and neurovascular coupling, which ensures neurons receive adequate oxygen and nutrition. Besides, microglia cooperate with astrocytes to modulate the neurotransmitter levels so as to prevent excitotoxicity by preventing neurotransmitter overloading (Cai et al., 2024). The expression of different types of cytokines and chemokines produced by microglia maintains the immune responses in the CNS, which guarantees immune homeostasis (Sadeghdoust et al., 2024). The permeability of the blood–brain barrier controlled by microglia was interactively realized by its interaction with endothelial cells within the blood–brain barrier. 20 Similarly, the blood–brain barrier’s permeability controlled by microglia interactively influences maintaining the internal environmental stability of the brain (Weng et al., 2024).

Synaptic pruning is a process necessary in neural development in which the activity of neural networks is sustained by eliminating aberrant or redundant synaptic connections (Hao et al., 2024). Complement proteins C1q and C3, which are surface expressed on microglia, can tag synapses for elimination via phagocytosis by microglia. Microglia also mold the synapse itself through partial phagocytosis or synaptic stripping (Cornell et al., 2022). It is one of the main mechanisms by which neural networks stabilize and become plastic. Microglia also secrete a multitude of neurotrophic factors involved in neuronal survival and regeneration (Gaire, 2022). Microglia have two phenotypes depending on the nature of inflammation: M1 (proinflammatory phenotype) and M2 (anti-inflammatory phenotype), which protect neurons from damage by secreting different cytokines that modulate the local inflammatory environment (Du et al., 2017). Microglia, upon activation or ischemia and subsequent brain injury, migrate to the injured site within a short time by phagocytosing dead cells and debris. Microglia are rapidly activated in response to brain injury or ischemia and migrate into the injured area to restore tissue and regenerate by removing dead cells and cellular debris. Microglia play a role in the regulation of energy metabolism by influencing glycolysis and oxidative phosphorylation that support normal neuronal function (Zhang G. et al., 2024).

### 2.2 Early diagnostic biomarkers

Biomarkers, imaging markers, and genetic markers are of crucial importance for the early diagnosis of AD. In biomarkers, low cerebrospinal fluid Aβ42, elevated total tau protein, and phosphorylated tau protein are typical markers for AD, and the latter two alterations occur in the prodromal stage of the disease and may be correlated with the severity of the disease (Andersson et al., 2025). Blood concentrations of neurofilament light chain are extremely elevated in early AD and correlate strongly with the severity of the disease and are also an early marker of diagnosis (Andersson et al., 2020). Exosomal miRNAs also exhibit an altered level of expression, the most notable of which is miR-15. Exosomal miRNAs are also abnormally expressed at early AD, and overexpression of miR-155 increases neuroinflammation and miR-124 inhibits inflammation and neuronal damage (Li Y. B. et al., 2024). Of the imaging markers, brain Aβ plaque and tau pathology positron emission tomography scanning and PET scanning using radiotracers reveal specific Aβ plaque binding and detect Aβ deposits at an early stage. Magnetic resonance imaging, however, reflects structural brain change, which is highly associated with cognitive decline (Mantzavinos and Alexiou, 2017). APOE ε4 allele is one of the most powerful gene risk factors of AD among the genetic markers, and allele carriers are greatly prone to early diagnosis (Di Battista et al., 2016). Gene mutations in TREM2 have been implicated in AD risk, and its absence in microglial cells disables Aβ clearance and neurologic inflammatory response (Heneka, 2023). CD33 gene polymorphism rs3865444 is associated with decreased AD risk and can be utilized in early diagnosis in the regulation of microglial Aβ clearance (Walker et al., 2015). Detection and verification of such markers are an excellent tool for early diagnosis of AD and enable early treatment and intervention of the disease (Li Y. B. et al., 2024).

### 2.3 Dysregulation of microglia in AD

Aβ has been reported to bind the TLR receptor on microglial surfaces and convert microglia to the M1-type phenotype (Thakur et al., 2023). These cytokines not only support neuroinflammation but also induce neuronal apoptosis. Aberrant activation of microglia also results in disruption of the blood–brain barrier, promoting increased peripheral immune cell infiltration (Cai et al., 2022; Versele et al., 2022; Sarb et al., 2024; van den Brink et al., 2024). Activation of microglia has been shown to trigger signaling cascades such as NF-κB and JNK via TNF-*α* specific binding to the receptors TNF-R1 and TNF-R2 (Wang D. et al., 2021; Wheeler et al., 2024). These signaling pathways increase the expression of other inflammatory cytokines that participate in pathological mechanisms such as induction of inflammation, apoptosis, and production of Aβ precursor protein and tau protein.

Under physiological conditions, microglia may phagocytose and maintain the homeostasis of Aβ in the brain. Microglial dysfunction contributes to a decreased ability to phagocytose Aβ and results in the accumulation of this protein in amyloid plaques within the AD brain. The released toxic substances by dysfunctional microglia include all kinds of oxygen radicals, nitrogen radicals, and glutamate (Salminen, 2024). It influences the stability and function of neural networks through modulating synaptic functions such as synaptic plasticity and synaptic density, leading to cognitive decline (da Fonseca et al., 2014; Wright et al., 2024). Abnormal activation of microglia promotes the infiltration of T cells, and through secretion of cytokines and direct cell contact driving hyperphosphorylation of tau proteins and the formation of neurofibrillary tangles (Tucsek et al., 2014; Sharallah et al., 2024), this exacerbates neurodegenerative pathology, putting extra emphasis on the multilevel pathological role of microglia in AD.

### 2.4 The influence of peripheral immune infiltration on AD

Peripheral immune cells can cross the central nervous system (CNS) through the blood–brain barrier (BBB) and communicate with microglia to regulate neuroinflammatory and neurodegenerative processes. In AD, Aβ plaque and tau protein tangle accumulation result in disruption of the BBB to make it permeable and provide an easy access to the CNS for peripheral immune cells (Zhang Q. et al., 2024). Microglia and astrocytes are activated simultaneously to secrete chemokines to invite peripheral immune cells into the CNS. Monocytes infiltrate the CNS and become macrophages that can phagocytose Aβ plaques but are hyper-activated to produce pro-inflammatory cytokines (Sun et al., 2024). B cells produce antibodies against Aβ and help clear Aβ plaques through the ADCC pathway. Peripheral immune cells and microglia exhibit competitive and synergistic interactions, for instance, macrophage and microglia can be synergistic while phagocytosing and degrading Aβ, yet the expression of TREM2 in macrophages can repress the action of TREM2 of microglia to affect the efficiency of Aβ clearance (Sharma et al., 2024). Various clinical trials indicated that fluctuations in blood monocyte count and T-cell subset are associated with cognitive impairment of AD patients, the therapeutic mechanism depending on peripheral immune cells, for instance, inhibition of the release of chemokines and modulation of the activity of T-cells, will be an innovative direction to treat AD in the future (Daraban et al., 2024).

### 2.5 Microglial polarization: protective vs. harmful phenotypes

The functional state of microglia, the principal immune cells of the CNS, is far more complex than the traditional M1 and M2 phenotype polarization. Current studies have shown that the state of microglia in AD can comprise a wide variety of different phenotypes that play different roles in different stages of the disease and microenvironments (Baligacs et al., 2024). Aside from the classic M1 and M2 phenotypes, studies have characterized conditions such as disease-associated microglia (DAM) and reactive microglia (RAM) that have specific functional and molecular profiles in AD pathology (Lana and Giovannini, 2025).

M1 microglia are activated when stimulated by proinflammatory factors such as interferon-*γ* and lipopolysaccharide, and release proinflammatory factors such as TNF-*α*, IL-6, IL-1*β* and inducible nitric oxide synthase. Excessive secretion of these factors aggravates neuroinflammatory reaction and toxic injury of neurons, and promotes the accumulation of Aβ and the hyperphosphorylation of tau protein (Darwish et al., 2023; Bhardwaj et al., 2024). In contrast, M2 microglia are activated by anti-inflammatory factors such as interleukin −4 (IL-4) and interleukin −13 (IL-13), and secrete anti-inflammatory and neurotrophic factors such as interleukin −10 (IL-10), transforming growth factor β (TGF-β), BDNF and glial-derived neurotrophic factor (GDNF) (Mirarchi et al., 2024; Table 1).

**Table 1:** The transition mechanisms and related factors of microglia between neuroprotective and neurotoxic states in AD.

Stage	Trigger factors	Transition direction	Related mechanisms	Outcome
Initial activation	Aβ deposition	From resting to activated state	Aβ binds to TLR receptors, activating NF-κB and JNK signaling pathways	Promotes Aβ clearance and release of inflammatory factors
Neuroprotective state	Anti- inflammatory factors	From activated to M2 phenotype	Activation of JAK/STAT/SOCSsignaling pathway, promoting anti- inflammatory and neurotrophic factor secretion	Promotes Aβ clearance, supports neuronal survival and repair
Neurotoxic state	Persistent Aβ stimulation, inflammatory factors	From activated to M1 phenotype	Sustained activation of NF-κB and NLRP3 inflammasome pathways, releasing pro- inflammatory factors	Exacerbates Aβ deposition, induces neuronal apoptosis and synaptic loss
Gene variation impact	Variations in TREM2, CD33, CR1, etc.	Depends ongene variation type	Gene variations affect microglial activation status and Aβ clearance capability	Enhances or impairs Aβ clearance, influences inflammatory response
Disease progression	Chronic inflammation, Aβ and tau pathology	From neuroprotective to neurotoxic	Sustained inflammatory responses and Aβ accumulation lead to microglial exhaustion	Neurodegenerationworsens, cognitivedecline

Additionally, disease-associated microglia (DAM) have distinct initial AD gene expression patterns and are found surrounding Aβ plaques and clear Aβ as well as modulate tau pathology (Martins-Ferreira et al., 2025). TREM2 variants were significantly associated with AD risk increase and that its physiological function is to allow for DAM formation and thus increased Aβ clearance (Wang H. et al., 2023). Pathology of tau also significantly increases with deficient TREM2 function or microglial deficiency, pointing toward an essential role of DAM in the prevention of tau spreading. The phenotypes of AD in microglia are beyond the simple M1 and M2 phenotypes but more evolved phenotypes such as DAM (Wang et al., 2025). Each state has its own corresponding functions to perform in the different stages of the disease and microenvironment, and in the future, further molecular mechanisms and functional difference among the states would have to be investigated by studies to un-scramble the multi-functional role of microglia in AD (Gao et al., 2023).

## 3 Microglial activation and AD pathogenesis

### 3.1 Early microglial activation and Aβ plaque formation

Microglial activation is immediately correlated with Aβ plaque development during the early stages of AD. Numerous studies have confirmed that microglia can identify Aβ through multiple pattern recognition receptors (Wu et al., 2024). In addition to mediating the identification of Aβ, these receptors also cause microglia to migrate toward where Aβ deposition occurs and are activated for phagocytosis by triggering downstream signal pathways (Zou et al., 2024). Microglia regulate local inflammation by secreting inflammatory and chemokine factors, which have the potential to affect the clearance and deposition efficacy of Aβ (Streit et al., 2024; Tournier et al., 2024). M2-type-activated microglia execute anti-inflammatory and reparative actions that are beneficial for Aβ clearance. In addition, disease-associated microglia (DAM) accumulate in the vicinity of Aβ plaques and are involved in Aβ clearance modulation and tau pathology. Dysregulated expression of the TREM2 gene, highly associated with increased risk of AD, can drive the formation of DAM to enhance Aβ clearance under physiological conditions. Pathologically stimulated microglia may result in overexuberant inflammatory responses and complement system activation, hence encouraging Aβ deposition and neuronal damage (Madhu et al., 2024; Rodriguez-Vieitez et al., 2024). Investigations in AQP4-deficient mice models have provided evidence that overactivation of microglia is strongly associated with Aβ clearance defect and Aβ deposition. Reducing microglia or inhibiting APOE expression could significantly affect Aβ deposition and clearance (Sethi et al., 2024).

### 3.2 Chronic inflammation and disease progression

Among the pathologic processes of AD, neuroinflammatory response is widely accepted to be one of the significant mechanisms that are instrumental in disease facilitation. Activated microglia and the cytokines secreted by them have been shown to be one of the major drivers of neuroinflammation in AD, since microglia are the resident immune cells of the CNS (Sian-Hulsmann and Riederer, 2024; Sobue et al., 2024; Yu et al., 2024). The majority of studies indicated that inflammatory cytokine expression, such as IL-1β, TNF-*α*, and IL-6, was highly upregulated in the brains of AD patients and were secreted predominantly by activated microglia (Shen Y. et al., 2024). In addition, IL-1β, TNF-α, and IL-6 not only increased neuroinflammatory responses but also exhibited direct toxic effects on neurons, inducing neuronal apoptosis and synapse loss (Liu X. H. et al., 2024; Ohm et al., 2024). It was established that Aβ peptide induced higher secretion of cytokines by microglia, supporting the perception that Aβ plays a considerable role in activating microglia (Huang et al., 2024; Kempuraj et al., 2024; Liu D. et al., 2024). This sets a feedback cycle of continuous release of cytokines, establishing a state of chronic neuroinflammation (Botella Lucena and Heneka, 2024; Cafferata et al., 2024; Gasparotto et al., 2024). TLR signaling was established as the regulator of the innate immune response in microglia and astrocytes, further corroborating the key role of cytokines in neuroinflammation (Ana, 2024).

The activation state of microglia is closely related to neurotoxicity in AD. Relevant studies have noted that the activation of microglia is very close to the Aβ deposition, neurofibrillary tangle formation, and neurodegeneration (Yang G. et al., 2023; Yang Y. et al., 2023; Zhou et al., 2023). Different pathological stimuli can influence changes in motility, morphology, phagocytosis, and release of cytokines, chemokine, reactive oxygen species, and prostaglandin metabolites in activated microglia (Maki et al., 2023). These changes allow microglia to turn into a neurotoxic phenotype, which release inflammatory mediators, leading to an enhancement of neuronal damage (Ma et al., 2023). The gene expression profiling has noted characteristic gene expression profiles with microglia that associate with Aβ plaques; moreover, gene expression was reduced with the steady state microglia (Le et al., 2023). This means that the activation of microglia not only correlates with the severity of neurodegeneration but could possibly contribute to disease development due to changes in gene expression (Demuth et al., 2023). More than normal TNF-*α* and IL-1β release closely relates to neuronal apoptosis and synaptic loss that enhance neurotoxicity (Bivona et al., 2023). In the case of phagocytosis of Aβ, if the microglia get overactivated, it may result in normal neuronal damage.

### 3.3 Microglia as mediators of Aβ and tau pathology

Microglia, which are central nervous system immune cells, are not only involved in the generation and clearance of Aβ plaques, but also perform the propagation and transmission of tau protein by a myriad of mechanisms (Jiao et al., 2024). Recent research has found that microglia over-secret extracellular vesicles (EVs) containing phosphorylated tau proteins in the vicinity of Aβ plaques, and that EVs transmit tau proteins between neurons (Al-Thani et al., 2024; Fan et al., 2024). This mechanism is responsible for the correlation between Aβ deposition and tau pathology, where the deposition of Aβ plaques activates microglia to secrete more EVs to promote tau protein spread at an accelerated rate (Arber et al., 2024; Fu et al., 2024; Tsering et al., 2025). Studies have shown that when TREM2 function is normal, microglia are able to effectively limit the seeding and spreading of tau protein around Aβ plaques, whereas tau pathology is significantly increased with TREM2 loss of function or microglia depletion (Kloske et al., 2024; Roveta et al., 2024). In the early stages of AD, microglia mainly maintain homeostasis, whereas in the later stages of the disease they are activated and transformed into disease-associated microglia (DAM) (Kanuri and Sirrkay, 2024; Kim et al., 2024).

**Figure 1:**
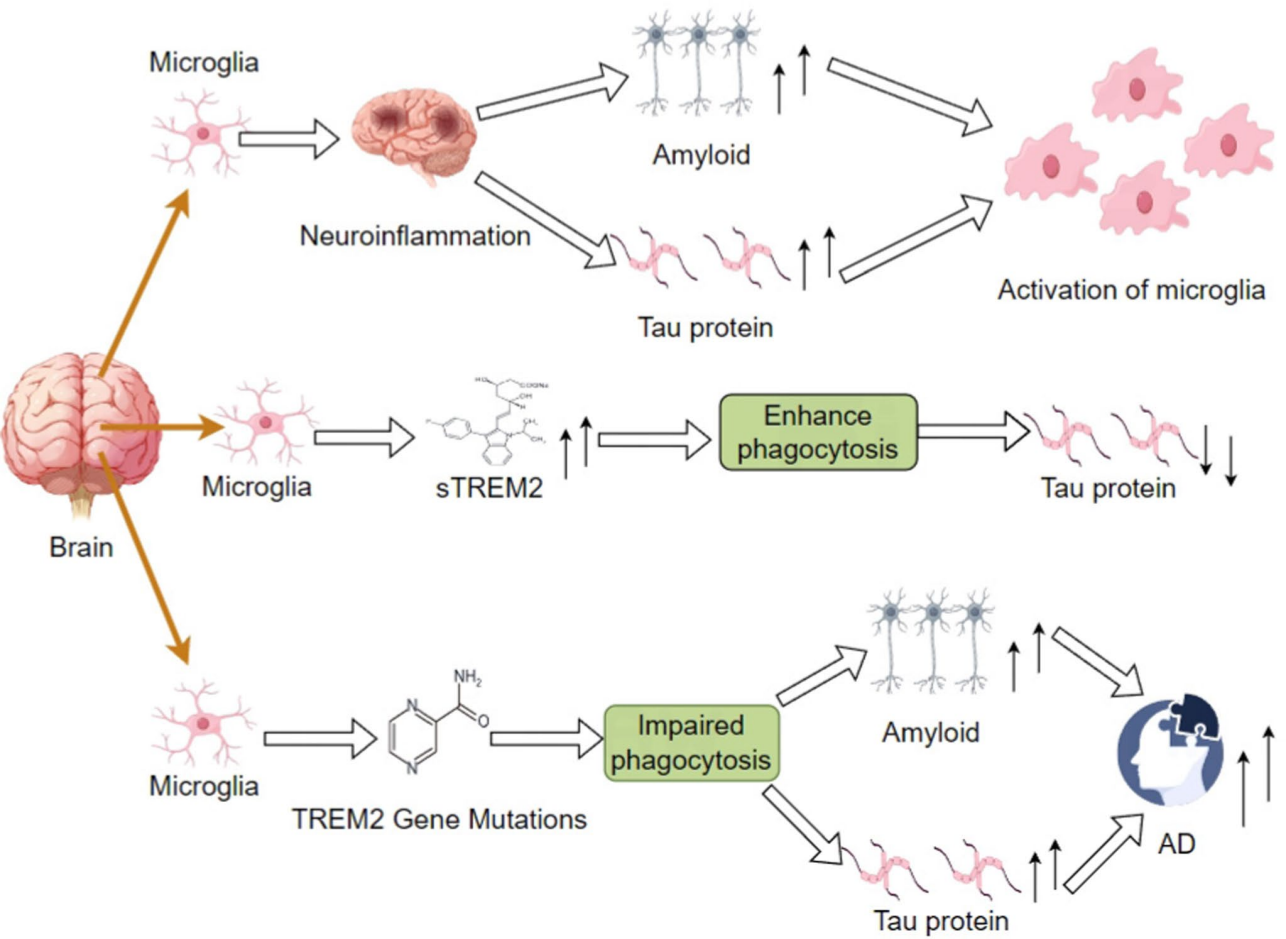
Differential impacts on amyloid and tau protein accumulation in AD via impaired phagocytic function by increased sTREM2 levels or TREM2 mutations; these may outline the mechanisms involved in neuroinflammation and the subsequent course of disease development.

A different study has shown that TREM2 gene variants strongly associate with AD risk and that TREM2 dysfunction results in microglia’s inability to clear Aβ plaques and tau proteins effectively (Ameli Mojarad and Ameli Mojarad, 2024; Pocock et al., 2024). Aβ plaque promotes phosphorylation and aggregation of tau proteins and neuroprogenitor fiber tangles formation (Chandra and Vassar, 2024). Deposition of Aβ has been shown to increase tau accumulation in studies via mechanisms involving microglia. In a model of microglia depletion, tau pathology near Aβ plaques was significantly increased, suggesting that microglia in general are suppressive of tau pathology (Kaur et al., 2024; Shahidehpour et al., 2024). Microglia over-produce EVs near Aβ plaques, not only facilitating the spreading of tau proteins but also aggravating neuroinflammation. Because microglia have a pivotal role in Aβ and tau pathology, it would be advantageous to diminish Aβ and tau pathology by stimulating TREM2 or augmenting induction of microglia to the DAM phenotype (Bathe et al., 2024; Figure 1).

### 3.4 Resolving the contradictions in microglial activation and tau pathology

Even though significant progress has been made in understanding the contribution of microglia to Aβ and tau pathology, literature on the link between microglia activation and tau pathology is conflicting, where some studies point toward it predisposing toward tau pathology induction while others suggest it may play a protective role against tau clearance, and these discrepancies may be owing to variations in experimental models, design, and the phase of the disease studied (Baligacs et al., 2024). In order to eliminate these discrepancies, future studies need to focus on the following. To resolve these contradictions, future studies would need to focus on the following aspects: first, normalizing experimental models and using consistent and well-characterized cell lines and animal models to reduce the impact of model variation (Anand et al., 2022); second, conducting longitudinal studies to follow the dynamic evolution of microglia activation and tau pathology with time and determine their functions at different stages of the disease (Wang C. et al., 2022); and third, conducting in-depth mechanistic studies to investigate the relationship between microglia activation and tau pathology (Chen X. et al., 2024). Third, comprehensive mechanistic studies will be conducted to investigate the molecular mechanism of the interaction between microglia activation and tau pathology; fourth, integration of data will be conducted, using meta-analyses and systematic reviews to aggregate data from multiple studies and identify common trends and variations; and fifth, advanced imaging tools will be utilized to visualize spatial and temporal correspondence between microglia activation and tau pathology *in vivo*, providing more intuitive evidence (Wu et al., 2025).

### 3.5 The role of microRNA in microglial activation

MicroRNAs are small non-coding RNAs that regulate gene expression by binding to the 3′ untranslated region of target mRNAs, inhibiting their translation, or directly degrading them. miRNAs can regulate key signaling pathways in microglia, affecting the inflammatory response and Aβ clearance of microglia. miRNAs in microglia regulate the inflammatory response and Aβ clearance of microglia by modulating key signaling pathways. miR-155 overexpression promotes microglial polarization to the pro-inflammatory M1 phenotype and NF-κB signaling pathway activation by targeting SHIP1 to promote the release of inflammatory factors (Zhang et al., 2025). The expression of miR-34a is increased in AD patients’ microglial cells, and the targeting of SIRT2 is enhanced in microglia. In microglia of AD patients, miR-34a expression is up-regulated and targets SIRT1 to inhibit its expression, affecting microglia metabolism and inflammatory responses, and thus Aβ clearance and neuroinflammation (Jadhav, 2024). Overexpression of miR-21 targets PTEN to stimulate the PI3K/Akt signaling pathway, promoting inflammatory responses and thus exacerbating neuroinflammation. Unlike miR-155, miR-124 inhibits M1-type polarization and promotes anti-inflammatory M2-type polarization to reduce neuroinflammation and neuronal injury. miR-124 suppresses the secretion of pro-inflammatory mediators and induces autophagy in the inflammation pathogenesis to alleviate neuroinflammation via targeting p62/p38 (Chen Y. et al., 2024).

## 4 Microglia and Aβ: mechanisms of clearance and toxicity

### 4.1 Microglial phagocytosis of Aβ and its impairment in AD

TREM2, which is highly expressed on the surface of microglia, interacts directly with Aβ through regulation of microglia function (Shi et al., 2024). Several studies have shown that TREM2 recognizes and binds to Aβ, promoting phagocytosis of Aβ from microglia. TREM2 activation enhances Aβ phagocytosis by microglia through many molecular pathways (Serrano-Pozo et al., 2021; Cosma et al., 2023; de Gea et al., 2023). TREM2 activation enhances phagocytosis in microglia by several molecular pathways, thus promoting Aβ clearance (Benitez et al., 2021). TREM2 activation promotes phagocytosis in microglia. TREM2 activation enhances the expression of various genes related to phagocytosis and promotes the phagocytic function of microglia. TREM2 forms complexes with APOE and CLU/APOJ apolipoproteins, regulates metabolic-state regulation of microglia, along with its autophagy function, thus maintaining its phagocytic and survival maintenance (Dvir-Szternfeld et al., 2022). Besides participating in Aβ phagocytosis and its clearance, the role of TREM2 goes to taking part importantly in the modulation of microglia chemotaxis, survival, and proliferation, with the purpose of maintaining their functionality (Gratuze et al., 2021; Murakawa-Hirachi et al., 2021). More recently, the role of a loss of function in TREM2 leading to mTOR pathway impairment in microglia, which has been implicated as having a main function in the autophagic process, apart from a role in cell metabolism. Indeed, lifetime risks of AD for carriers of TREM2 loss-of-function mutations are remarkably increased, rivaling or even surpassing those for carriage of APOE ε4 variants (Burton et al., 2024; Escamilla and Salas-Lucia, 2024; Etxeberria et al., 2024). Such findings underscore the critical role of TREM2 in maintenance of healthy microglia and Aβ clearing.

Loss or impairment of TREM2 function has been associated with a significant reduction of microglia phagocytosis (Giorgio et al., 2024). In the TREM2-deficient 5 × FAD mouse model, there is an increased ratio of lipidated LC3II to nonlipidated LC3I with increased numbers of autophagic vesicles, indicating impaired process of autophagy (Zhong et al., 2024). TREM2 may be considered the central regulator for phagocytosis in microglia. Its activation promotes the phagocytosis and degradation of Aβ by microglia (Zhang W. et al., 2024). Moreover, phagocytic dysfunction has affected the efficiency of Aβ clearance and promoted Aβ accumulation by several pathways. Moreover, it has been shown that the activation of the NLRP3 inflammasome in AD is associated with the deposition of Aβ, which may result from an inflammatory response due to an impaired phagocytosis process in microglia. Microglial-related studies implicate that impaired phagocytosis mediates Aβ production through NF-κB signaling pathways and increases in BACE1 expression, which eventually lead to accumulation in Aβ within brain tissue and hastening of the AD pathological course (Wang et al., 2024; Yan et al., 2024).

### 4.2 Microglial dysfunction and Aβ deposition

Microglia trigger downstream inflammatory signaling cascades by detecting Aβ aggregates through recognition receptors. Aβ42 activates the TRPM2 channels by inducing oxidative stress, resulting in pro-inflammatory cytokine release, and is a positive feedback loop to enhance the inflammation process (Shen Z. et al., 2024). Aβ has the ability to also activate the NLRP3 inflammasomes of microglial cells to cleave caspase-1 and mature IL-1β. This is amplified on microglial inflammation, initiating a negative chronic inflammatory state leading to necrosis to the neurons (Zhang L. et al., 2024). Activation of binding between TREM2 and Aβ initiates proliferation, phagocytosis, and secretion by microglia, modulating their metabolism and viability. Despite being protective, overexposure to Aβ has been shown to lead to microglial dysfunction, reduced clearance, and perpetuation of relentless inflammatory signaling (Wang M. et al., 2022).

Aβ-activated microglia produce proinflammatory cytokines such as TNFα and IL-1β that induce excitotoxicity and neuronal death via the synergistic activation of neuronal TNF receptors and NMDA receptors. This neuroinflammatory cascade response contributes to synaptic dysfunction and neuronal loss in AD (Shi et al., 2022). While microglia phagocytosis is critical in Aβ clearance, sustained Aβ exposure may lead to their functional depletion as evidenced by dendritic shortening, reduction in coverage area, and fragmentation of cellular processes suggestive of loss of surveillance function. Functionally impaired microglia lose the capability of effective Aβ clearance, which consequently accumulates in the brain and promotes AD pathology (Bandyopadhyay, 2021; Wang S. Y. et al., 2021). In cellular models of AD, Aβ-treated microglial cells showed an impairment of autophagy as defined by reduced expression of the autophagy-related protein Beclin-1. Defective autophagy disturbed the degradation capability of microglia for Aβ, resulting in Aβ accumulation and enhancement of inflammatory responses (Katsumoto et al., 2018).

### 4.3 Genetic variants affecting microglial Aβ clearance

#### 4.3.1 TREM2

TREM2 is constitutively strongly expressed on microglia in the central nervous system and regulates microglia phagocytosis and signaling through interaction with DAP12 protein. TREM2 directly deoves Aβ oligomers and activates the downstream PI3K/Akt signaling pathway, and facilitates Aβ degradation and phagocytosis by microglia. In the initial stage of AD, TREM2 activation enhances the chemotactic activity of microglia to migrate to the site of Aβ deposition and clear plaques to play a neuroprotective role. TREM2 loss-of-function mutations result in defective phagocytosis of microglia and significant reduction in the effectiveness of Aβ clearance, as well as suppression of secretion of the anti-inflammatory factor IL-10 to further amplify chronic neuroinflammation (Li et al., 2022). TREM2-deficient mice have been shown to be unable to encapsulate Aβ plaques in mouse models, leading to synaptic loss and hyperphosphorylation of tau proteins of neighboring neurons. TREM2 maintains long-term survival of microglia by controlling their metabolism, and its dysfunction may lead to microglia depletion, which could exacerbate Aβ deposition and neurodegeneration (Lin et al., 2024).

#### 4.3.2 CD33

CD33 is a salivary acid-binding immunoglobulin-like lectin whose expression level dictates risk for AD by controlling immune checkpoint activity in microglia. rs3865444 CD33 gene polymorphism reduces the expression of intact CD33 but enhances D2-CD33 that lacks exon 2. D2-CD33 enhances microglial cell, via the ITAM signaling pathway, Aβ phagocytosis as well as inflammatory inhibitory factor secretion (Javor et al., 2022). Microglia in subjects with the rs3865444 minor allele are enhanced in Aβ clearance and inhibited in the release of proinflammatory factors. On the other hand, full-length CD33 overexpression inhibits microglia polarization into the M2 subtype and leads to defective Aβ clearance and ongoing neuroinflammation, proving the crucial role of CD33 splicing homeostasis in regulating microglia immune homeostasis (Li et al., 2015).

#### 4.3.3 CR1

CR1 is expressed primarily on the surface of microglia and facilitates Aβ coagulation and precipitation by binding to Aβ, thereby facilitating phagocytosis and clearance of Aβ by microglia. CR1 gene polymorphisms are strongly associated with AD susceptibility, affecting the expression level of the CR1 protein and microglial efficiency of Aβ clearance (Sudwarts and Thinakaran, 2023). CR1 gene variants affect the condition of microglial activation and inflammatory reaction, causing bias toward M1-type activation and suppressing their Aβ clearance. CR1 gene variants suppress Aβ clearance through affecting the condition of microglial activation and inflammatory reaction, causing bias toward M1-type microglial activation (Zuroff et al., 2017).

#### 4.3.4 APOE

APOE gene polymorphisms tightly control AD pathology by modulating lipid metabolism and inflammatory processes in microglia. APOEε4 has a higher affinity to Aβ than APOE2/3 but, via its defective binding to lipoproteins, causes reduced phagocytosis of Aβ by microglial cells. APOEε4 also induces mitochondrial damage in microglial cells and increases ROS production and NLRP3 inflammatory vesicle activity (Qu et al., 2024). APOEε4 blocks the conversion of microglia into a neuroprotective phenotype, induces the release of proinflammatory extracellular vesicles, and promotes the transneuronal transmission of tau proteins by blocking the TREM2-PI3K/Akt signaling pathway, demonstrating that APOE genes are importantly involved in the pathomechanisms of AD (Narasimhan et al., 2024).

#### 4.3.5 PLCG2

It was identified that PLCG2 is up-regulated selectively in plaque-associated microglia and engaged in the regulation of microglia phagocytosis and inflammation response. These variants bi-directionally control microglia phenotype and function affecting Aβ clearance competency through activating divergent transcriptional programs. These results not only indicate the widespread involvement of PLCG2 in AD pathogenesis, but also infer that it could be a prospective therapeutic target for future AD treatment (Magno et al., 2019; Staley et al., 2024).

#### 4.3.6 ABI3

The ABI3 gene encodes a transcription factor with a B3 domain and is at the center of microglial immune responses. Some variants, such as p.S209F, which was significantly associated with LOAD risk, may alter the efficiency of Aβ clearance by microglia through their impact on ABI3 function. Such variants lead to impaired activation and phagocytosis of microglia, thus affecting Aβ clearance and exacerbating pathological progression of AD (Ibanez et al., 2022; Smith et al., 2022; Table 2).

**Table 2:** Genetic variants and their impact on microglial function and Alzheimer’s disease risk.

Genetic Variant	Functional Impact	Association with AD Risk	References
TREM2	TREM2 gene mutations may lead to microglial dysfunction, affecting their clearance of Aβ	TREM2 gene mutations areassociated with the exacerbation of AD pathological processes	Li et al. (2022)
CD33	The rs3865444 polymorphism leads to a reduction in full-length CD33 and an increase in D2-CD33, enhancing microglial Aβ clearance function	The minor allele of rs3865444 is associated with a reduced risk of AD	Javor et al. (2022)
CR1	Specific CR1 gene variants affect CR1 expression and function on microglia, regulating Aβ clearance efficiency	CR1 gene variants are significantly associated with the risk of LOAD	Sudwarts and Thinakaran (2023)
APOE	The APOE4 allele hasa higherAβ binding affinity compared to APOE3 and APOE2, leading to reduced Aβ clearance efficiency	The APOE4 allele plays a key role in the pathologicalprogression of AD	Narasimhan et al. (2024)
PLCG2	PLCG2 gene variants, especially the P522R polymorphism, may improve Aβ clearance and neuroinflammatory responses	The P522R variant has a protective effectin LOAD	Magno et al. (2019)
ABI3	Specific gene variants, such as p.S209F, may alter microglial clearance efficiency of Aβ	ABI3 gene variants are significantly associated with the risk of LOAD	Smith et al. (2022)

## 5 Genetic and molecular regulation of microglia in AD: implications for neuroimmune interactions

### 5.1 Molecular pathways regulating microglial activation

At the initial phase of AD, TREM2-PI3K/Akt pathway dominates, and increased Aβ clearance and inhibition of NF-κB can delay pathological development (Hu et al., 2025). At the advanced phase of AD, continuous activation by Aβ leads to TREM2 decrease, decreased PI3K/Akt activity, excessive activation of NF-κB and exuberant release of inflammatory cytokines enhance tau pathology spreading and neuronal loss (Zhang J. et al., 2024). TREM2 mutations lead to the disturbance of PI3K/Akt-NF-κB homeostasis, causing microglial cell dysfunction and exacerbation of AD (Sun et al., 2021).

#### 5.1.1 TREM2 signaling pathway

TREM2 was found to mediate the process in the central nervous system by binding to a series of ligands, such as Aβ oligomers and phospholipids, by transmitting the downstream DAP12/DAP10-SYK-PI3K-AKT signaling cascade to regulate microglia phagocytosis, survival, inflammatory response, and metabolic processes (Lin et al., 2024). TREM2 activation facilitates microglia phagocytosis and enhances Aβ degradation by facilitating the upregulation of phagocytosis-related genes via the PI3K/Akt pathway. TREM2 inhibits nuclear translocation of NF-κB via the PI3K/Akt pathway and reduces secretion of proinflammatory factors to inhibit chronic neuroinflammation. TREM2-PI3K/Akt signaling modulates microglia energy metabolism and maintains their long-term survival and functional homeostasis (Yao et al., 2019).

#### 5.1.2 NF-κB signaling pathway

NF-κB signaling pathway is the key regulator of inflammatory and immune responses of microglia. TREM2, which is one of the highly expressed microglia-restricted receptors, was discovered to exert influence on the production of inflammatory factors by inducing downstream NF-κB signaling upon association with DAP12. Activation of TREM2 inhibits NF-κB-induced production of inflammatory factors to mitigate neuroinflammation. These observations demonstrate an immunomodulatory function of TREM2 in AD and a critical role for the NF-κB pathway in microglial activation. TREM2-activated PI3K/Akt pathway inhibits excessive inflammation by phosphorylating IκB kinase, blocking IκBα degradation and inhibiting NF-κB nuclear translocation (Shi and Huang, 2023).

#### 5.1.3 NLRP3 inflammasome pathway

Being a cytoplasmic multiprotein complex, NLRP3 inflammasome senses endogenous danger signals and pathological protein aggregation. Activation of NLRP3 inflammasome further promotes the maturation and secretion of proinflammatory cytokines IL-1β and IL-18, which further enhance neuroinflammatory responses and drive neurodegenerative processes. Aβ deposition directly activates the NLRP3 inflammatory vesicles in microglia, enhancing the secretion of IL-1β and further exaggeration of neuroinflammation and neuronal injury with positive feedback (Hanslik and Ulland, 2020; Zhang Z. et al., 2024).

#### 5.1.4 PI3K/Akt signaling pathway

Microglia are implicated in diverse neurobiological processes by activating the PI3K/Akt signaling pathway in AD pathology. Experiments demonstrate that activation of the PI3K/Akt pathway suppresses the release of pro-inflammatory mediators and enhances the release of anti-inflammatory mediators that regulate AD-associated neuroinflammation. Activation of the PI3K/Akt pathway suppresses GSK3β activity in microglia and diminishes Tau protein phosphorylation and neurofibrillary tangle formation (Long et al., 2021). PI3K/Akt promotes M2-type polarization of microglia and inhibits M1-type polarization and immune microenvironment balance. PI3K/Akt regulates the glycolysis and oxidative phosphorylation of microglia through mTORC1, which modulates their clearance function and inflammatory phenotype. TREM2 significantly enhanced microglial cell neuroinflammation by activating the PI3K/Akt/GSK3β pathway, significantly enhancing spatial cognition in the APP/PS1 mouse model. These investigations represent the critical function of the PI3K/Akt pathway in regulating AD pathology and promise therapeutic targets (Chu et al., 2021).

#### 5.1.5 MAPK signaling pathway

Aβ acts on TLR4 on the microglial cell membrane to initiate the MAPK signal pathway, which phosphorylates heterogeneous downstream substrates like transcription factors and cytoskeletal proteins and modulates the synthesis of proinflammatory cytokines and chemokines. The MAPK signal pathway induces polarization of the microglial cells into the M1-type, augmenting the neuroinflammatory microenvironment of the brain and being harmful to the survival and functions of the neurons. MAPK signaling pathway also regulates polarization of microglia to the M2 form, which holds anti-inflammatory as well as neuroprotective function against neuronal damage. Balance in between the two polarized forms, i.e., the M1 or the M2 type, decides outcomes of disease and interruption of MAPK signaling pathway would theoretically result in interruption in balance toward excessively pro-inflammatory track (Wang C. et al., 2023). Confirmatory experiments have also shown that etomidate enhances cognitive function and suppresses oxidative stress and inflammatory response in AD mice brain tissue through regulation of the MAPK/ERK signaling pathway. The results indicate that inhibitors of the MAPK signaling pathway are likely to be potential drugs in the treatment of AD (Zuppe and Reed, 2024; Table 3).

**Table 3:** Overview of key signaling pathways involved in microglial activation and their roles in Alzheimer’s disease pathology.

Signaling pathway	Functional description	Related Molecules/Ligands	Effects after pathway activation	References
TREM2 signaling pathway	Regulates microglial phagocytosis, cell survival, inflammatory responses, and metabolism	Aβ oligomers, phospholipids	Activates the downstream DAP12/DAP10- SYK-PI3K-AKT signaling axis, thereby modulating microglial functions	Yao et al. (2019)
NF-κB signaling pathway	Regulates microglial inflammatory and immune responses	TNF-α, IL-1β	Activation affects the production of inflammatory factors and interacts with TREM2	Shi and Huang (2023)
NLRP3 inflammasome pathway	Detects endogenous danger signals and pathological protein aggregation, promoting the maturation and secretion of IL-1β and IL-18	Aβ, IL-1β, IL-18	Activation enhances the secretion of IL-1βand IL-18, forming a positive feedback loop that exacerbates neuroinflammation and neuronal damage	Zhang Z. et al. (2024)
PI3K/Akt signaling pathway	Involved in various neurobiological processes in the pathology of AD	GSK3β, AKT1	Activation inhibits GSK3β activity in microglia, reducing tau protein phosphorylation and neurofibrillary tangle formation	Chu et al. (2021)
MAPK signaling pathway	Regulates microglial activation and polarization states, with the MAPK pathway playing a pivotal role	p38, JNK	Activation amplifies neurodegenerative changes observed in AD	Zuppe and Reed (2024)

### 5.2 Neuroimmune modulation: genetic insights into AD therapy

Gene therapy technology also shows much potential in AD treatment studies. By utilizing gene editing techniques like CRISPR/Cas9, it is possible to directly regulate the expression level of genes associated with AD in an effort to enhance microglia function. From studies, microglia-induced neuroinflammatory responses could be significantly diminished by regulating TREM2 and CD33 genes. Small molecule drugs also regulate the inflammatory response of microglia (Garcia-Gonzalez et al., 2024). The P2X7 receptor inhibitors and the NLRP3 inflammatory vesicle inhibitors have been shown to inhibit the inflammatory response of microglia. They inhibit the release of inflammatory mediators and eliminate the threat of damage and death in neurons by controlling the signaling pathways involved selectively (Doshi et al., 2024). Their activities provide new hope for pharmacologic treatment of AD, and show promising activity against microglia-induced neuroinflammation.

### 5.3 Challenges and limitations of microglial targeted therapies

Though microglia-directed therapeutic interventions for AD have shown promise, there are still numerous areas where clinical translation is problematic. Drug delivery effectiveness is limited by the blood–brain barrier, and although nanocarriers, targeted ultrasound, and other platforms are used to enhance penetration, their safety, stability, and scale-up remain open questions, and dynamic change in expression of microglial cell-specific receptors reduces targeting efficacy (Liu et al., 2022). Inadequate targeting and functional/molecular marker overlap between microglial cells and peripheral macrophages may lead to drug Misuse of the peripheral immune system to cause systemic inflammation or immunosuppression (Baumgartner et al., 2025). Clinical trial failures are not unusual, and several clinical trials for neuroinflammatory disorders have failed to meet expected endpoints. Experiments have shown that although the anti-Aβ monoclonal antibody Aducanumab activates microglia to clear plaques, its contentious efficacy and side effects such as cerebral edema highlight the importance of balancing immune activation and neuroprotection (Niazi, 2024). Drugs that block NLRP3 inflammatory vesicles reduce tau pathology in animal models but have limited effectiveness in humans due to the activation of compensatory pathway activity or off-targeting (Zhou L. et al., 2024).

The second main challenge is the functional changes in microglia during aging. Microglia are morphologically, phenotypically, and functionally modified in brain aging, and such changes may impact their response to therapeutic intervention. With age, microglia experience alterations including diminished proliferative and phagocytic function, as well as increased basal inflammatory activity, and these alterations can modulate the activity of microglia in order to maintain neuronal homeostasis (Antignano et al., 2023). As age progresses, increased blood–brain barrier permeability can impact drug delivery and distribution to microglia. Microglial heterogeneity of the aging brain also makes it challenging to develop microglia-specific treatments, as different subpopulations of microglia may respond differently to treatment. Therefore, IAG-dependent microglial changes should be considered when creating and evaluating treatments in AD (Stamataki et al., 2024).

## 6 Conclusions and perspectives

During the last decade, microglial activation has been a pivotal driving force of AD pathogenesis, and therapeutic intervention in engaged signaling pathways has emerged as a novel therapeutic approach. Gene therapy and small-molecule drugs have been shown to possess the capacity to reduce inflammation and neuroprotection, but their clinical utility remains handicapped by challenges of targeting efficacy, pharmacokinetics, and long-term safety. Future and ongoing studies should examine deeper the microglia signaling pathways and design new drugs that guarantee safety, efficacy, and person-specific adaptability of the new treatment. We should further utilize single-cell sequencing technology to intensively investigate the various subpopulations of microglia in AD and their multiple functional differences under different phases of the disease; design nanocarriers or targeted ultrasound technology capable of crossing the blood–brain barrier for improving drug-delivery efficacy; study in particular the roles of peripheral immune cells in AD and design therapy with the goal to target microglia and peripheral immune cells simultaneously; and intensively investigate the role of miRNA in microglia activation and neuroinflammation. Cell activation and neuroinflammation, and devise miRNA-based therapeutic strategies; adopt more adaptive clinical trials, and utilize biomarkers for early diagnosis and evaluation of therapeutic effects; explore the combined modality of gene therapy and small molecule drugs, and explore the application of gene editing technology in microglia; perform longitudinal studies of dynamic changes of microglia at different stages of AD and explore their role in early diagnosis and control of disease; perform longitudinal studies of dynamic changes of microglia at different stages of AD and explore their role in early diagnosis and control of disease. Referring to their potential roles in early detection and monitoring of the disease. In these specific research directions, future studies may have a clearer understanding of microglia’s multi-faceted mechanisms in AD and establish a firm foundation for more effective therapeutic strategies.
